# Implementation of Physician Orders for Life Sustaining Treatment in Nursing Homes in California: Evaluation of a Novel Statewide Dissemination Mechanism

**DOI:** 10.1007/s11606-012-2178-2

**Published:** 2012-08-10

**Authors:** Neil S. Wenger, Judy Citko, Kate O’Malley, Allison Diamant, Karl Lorenz, Victor Gonzalez, Derjung M. Tarn

**Affiliations:** 1 UCLA Division of General Internal Medicine and Health Services Research, 911 Broxton Plaza #309, Los Angeles, CA 90024 USA; 2 Coalition for Compassionate Care of California, Sacramento, CA USA; 3 California HealthCare Foundation, Oakland, CA USA; 4 Greater Los Angeles VA Medical Center, Los Angeles, CA USA; 5 UCLA Department of Family Medicine, Los Angeles, CA USA; 6 UCLA Division of General Internal Medicine and Health Services Research, 911 Broxton Plaza, Los Angeles, CA 90024 USA

**Keywords:** nursing home, POLST, end-of-life care, community intervention

## Abstract

**BACKGROUND:**

Implementing Physician Orders for Life Sustaining Treatment (POLST) forms aims to improve communication of life-sustaining treatment preferences across care venues. California enabled this clinical tool in 2009, and a novel intervention of community coalitions was undertaken to advance POLST in localities around the state. Coalitions engaged facilities, including nursing homes (NHs), to foster POLST adoption. Eighteen months after introduction of POLST, we studied POLST implementation in California NHs.

**METHODS:**

NHs randomly selected in coalition and non-coalition areas were mailed surveys about POLST preparation and use in 2010. Coalitions identified which NHs they worked with.

**RESULTS:**

Of 546 NHs surveyed, 143 (52 %) in coalition areas and 141 (52 %) in non-coalition areas responded. In 82 % of responding NHs, staff received POLST education and 59 % of NHs reported having a formal policy on handling POLST. Two-thirds of NHs had admitted a resident with a POLST, and 15 % of newly admitted residents over the past month had a POLST (range 0–100 %). Eighty-one percent of NHs had completed a POLST with a resident. Fifty-four percent of residents were estimated to have a POLST (range 0–100 %) (coalition area NHs 60 % vs. non- coalition area NHs 48 %, *p* = 0.02). Within coalition areas, NHs that had worked with coalitions were more likely to have completed a POLST with a resident after admission than NHs that had not worked with coalitions. Few NHs (7 %) reported difficulty following POLST orders, but 38 % noted difficulty involving physicians in POLST completion.

**CONCLUSION:**

Less than 2 years after introduction, many California nursing homes report using POLST, although some NHs reported no experience. A novel community coalition intervention facilitated POLST implementation.

**Electronic supplementary material:**

The online version of this article (doi:10.1007/s11606-012-2178-2) contains supplementary material, which is available to authorized users.

## INTRODUCTION

Efforts to appropriately match medical care with patients’ goals for care focus on advance care planning—that is, discussing a patient’s clinical condition and prognosis and mapping out future care according to a patient’s preferences—and ensuring that such plans transition with patients across care venues to guide care. Despite broad endorsement of advance care planning,^1^ continuity of preferences across venues^2^^,^ 3 and over time^4^ has been poor, resulting in the potential for patients to receive treatments that they would not have desired. Because aggressive treatment is the default, misunderstood preferences translate into treatments not aimed at patient goals rather than symptom management.^5^ The Physician Orders for Life-Sustaining Treatment (POLST) paradigm facilitates elicitation of preferences for care from patients, and then ensures that these wishes are honored wherever the patient receives care.^6^ POLST captures preferences concerning cardiopulmonary resuscitation, overall aggressiveness of care, transfer, tube feeding, and perhaps other treatments. POLST can be a stimulus for healthcare providers to engage patients with serious, life-limiting illness in a discussion about preferences for intensity of care.^7^

The brightly colored form is signed by the patient (or proxy, if appropriate) and a clinician, and authority to follow the stated preferences is ensured by law or another mechanism, depending on venue. POLST, originally developed in Oregon^8^ and expanding rapidly,^9^ became effective in California in January 2009.

Prior work suggests that POLST improves advance care planning and translation of goals into care. A prospective analysis of nursing home patients with a POLST containing a do not resuscitate order and a desire for transfer only if comfort measures failed showed that over 1 year, no patient received resuscitation, intensive care unit care or ventilator support, and only 2 % were hospitalized to extend life.^8^ Similarly, among 58 decedents in a PACE program who had a POLST, use of resuscitation, antibiotics, intravenous fluids, and feeding tubes nearly always matched specified preferences.^10^ Surveys in venues employing POLST have revealed uptake by nursing homes^11^ and emergency medical technicians,^12^ and value in translating preferences into care.^13^

While such evidence suggests that POLST has the potential to improve end-of-life care, many of the advanced implementations of POLST (such as the state of Oregon and city of La Crosse, WI) have been in relatively delimited, socially homogeneous areas. Implementation of POLST in California presented a formidable task, given the size of the state and its ethnic diversity. In order to roll out POLST in California, the California HealthCare Foundation (a non-profit grant maker focused on improving healthcare in California), in concert with the Coalition for Compassionate Care of California (a statewide partnership promoting high-quality end-of-life care in California), developed a novel dissemination mechanism that employed community coalitions to perform grassroots education and training. This POLST implementation effort began in California in 2007, with creation of a statewide task force of stakeholders and funding of seven local grassroots coalitions to introduce POLST in their communities. The project, which focused initially on promoting POLST in nursing homes, had three strategies: to implement POLST in local geographic areas, to create a standardized approach to POLST implementation, and to engage stakeholders and regulators to sustain POLST implementation. In 2008, 11 additional community coalitions were funded, bringing the total to 18. As of January 2009, California law required that POLST be honored across settings of care, and provided immunity to providers who honor a POLST document in good faith.

We studied POLST implementation and use in nursing homes approximately 18 months after it was introduced in the state, in order to accomplish two objectives: (1) understand how the POLST paradigm disseminated early after its introduction, and (2) evaluate the effect of the novel implementation mechanism used in California.

## METHODS

We developed a questionnaire and surveyed nursing homes in community coalition and non- community coalition areas. The evaluation aimed to understand the structural changes and education undertaken by nursing homes to implement POLST, their experience with POLST use, and problems encountered. We analyzed survey responses in order to describe overall penetration of POLST use in California nursing homes, and to compare use between coalition and non-coalition areas, and among facilities within coalition areas.

### Nursing Home Survey Development

In collaboration with the California Association of Health Facilities (CAHF), we developed a survey instrument aimed to be completed by the director of nursing or an administrator. This survey (Appendix available online) asked about structural efforts and staff education to implement POLST, the percentage of residents admitted to the nursing home over the past 30 days who arrived with a completed POLST, percentage of current nursing home residents who had a POLST, and whether the nursing home had encountered specific problems with use of POLST.

The survey was developed in consultation with directors of nursing; items aimed at understanding problems with POLST implementation were based on interviews with nurses and nursing home physicians and administrators. The survey instrument was modified using cognitive interviews^14^ and then piloted (mean completion time: 9 min) with two respondents at each of 12 nursing homes. This revealed kappa levels between respondents in the range of 0.67 to 0.83, and correlation coefficients for continuous variables ranging from 0.77 to 0.92.

### Sample Specification and Survey Implementation

We selected a survey sample starting with a list of all nursing homes in California, excluding long-term acute care hospitals (LTACHs) and psychiatric facilities. We endeavored to obtain a representative sample of nursing homes from each of the coalition areas, and also a sample of nursing homes from analogous non-coalition areas. For the 18 coalitions, we selected the county of the coalition as the area from which to select nursing homes; in two of the cases, coalitions worked with nursing homes in more than one county and three coalitions in large urban areas worked in only a small area of the county. We identified 18 counties or portions of counties that were similar to coalition areas as comparison areas. Overall, the nursing home sample was selected from 32 counties that contained 94 % of the nursing homes in California (see Table 1).

**Table 1:** Nursing Home Study Sample*

Community coalition areas			Non-community coalition areas		
	N ^†^	Response		N	Response
Alameda/Contra Costa	19 (8)	8	Fresno	19	10
Humboldt/Del Norte	7 (6)	3	LA-East	20	12
Kern	15 (3)	10	LA-North	20	12
LA-SFV	20 (2)	11	LA-Long Beach	21	11
LA-West	20 (1)	8	LA-Pasadena	19	10
Mendocino	4 (4)	3	Lake	3	1
Monterey	16 (15)	11	Marin	16	3
Napa	5 (4)	1	Placer	11	7
Orange	20 (8)	8	Orange-East	15	6
Riverside	20 (9)	12	San Francisco	14	7
Sacramento	20 (2)	13	San Joaquin	20	14
San Bernardino	20 (10)	8	San Mateo	19	9
San Diego	18 (8)	7	San Luis Obispo	9	6
Santa Clara	20 (10)	10	Santa Barbara	13	6
Santa Cruz	10 (6)	6	Shasta	8	5
Sonoma	19 (10)	14	Solano	10	5
Ventura	18 (8)	6	Stanislaus	19	12
Yolo	6 (5)	4	Tulare	13	4
**TOTAL**	**277 (119)**	143 (51.6 %)	**TOTAL**	**269**	140 (52.0 %)

* Excludes nine nursing homes that were excluded because they were closed, psychiatric facilities or LTACHs (five in coalition counties and four in non-coalition counties)

^†^The “N” column indicates the number of nursing homes selected for survey in the area. For coalition areas, the number in parentheses designates the number of these selected nursing homes that the coalition listed as a facility with which they worked

In each coalition and non-coalition area, we selected 20 nursing homes, or if there were fewer than 20 nursing homes in the area, we selected all the nursing homes. For coalition areas, nursing home selection accounted for coalition reports of nursing homes they had worked with. In these coalition areas, we attempted to select half of the nursing homes from the coalition list and half that were not on their list. We randomly selected nursing homes to fill the complement of the coalition-listed and non-listed nursing home groups. For example, if a coalition is in an area that had more than 20 nursing homes and listed more than ten as having worked with them, then we randomly selected ten nursing homes in their area with which the coalition had not worked and ten nursing homes they had worked with. If there were an insufficient number of nursing homes that a coalition had worked with, then we backfilled from the group the coalition had not worked with, and vice versa. For non-coalition areas, we randomly selected up to 20 nursing homes. Overall, this process yielded 273 non-coalition nursing homes and 282 coalition area nursing homes, of which the coalitions had worked with 119.

Surveys were mailed in July 2010, with a cover letter signed by CAHF and the Coalition for Compassionate Care of California, and with a stamped envelope to return the survey to the investigators at UCLA. The survey was distributed as a web interface via e-mail by CAHF. Two rounds of reminder mailings were sent. In October, telephone calls were placed to request survey completion and to complete the survey by telephone, if desired. The study protocol was approved by the UCLA institutional review board (#10-001565).

### Statistical Analyses

We summarized survey responses to describe for the overall sample: nursing home structural and educational efforts concerning POLST, and residents admitted with and administered a POLST at nursing homes. We compared these responses between nursing homes in coalition and non-coalition areas using chi square tests and *t*-tests, as appropriate. We also present nursing home reports of issues in implementing POLST.

In order to better understand the impact of the community coalitions, we evaluated the survey responses of the nursing homes in their areas, accounting for the depth of interaction between the coalition and the nursing home. Prior to distribution of the survey, each coalition not only indicated with which nursing homes they had worked, but also the level of interaction with that nursing home, rated on a scale of 1 = low, 2 = moderate and 3 = high. Using these ratings, we evaluated the impact of coalitions on nursing homes by evaluating the relationship of the level of interaction (assigning 0 to nursing homes in the coalition area with which the coalition had not worked) with nursing home reports of POLST use. Within coalition areas, we compared nursing homes with which the coalition reported any interaction vs. those with no interaction using *t*- and chi square tests, and we evaluated the “dose–response” of nursing home reports to the level of coalition interaction using chi square and ANCOVA.

## RESULTS

Of the 555 nursing homes, nine were excluded (five in coalition areas and four in non-coalition areas) because they were a psychiatric facility, an LTACH or had closed. Among the 277 coalition area nursing homes, 143 responded (51.6 %) and among the 269 non-coalition nursing homes, 140 responded (52.0 %) (Table 1).

### POLST Education and Structural Changes

Nearly all nursing homes indicated there was a designated place to keep a POLST form, 70 % had a POLST “champion,” and more than half indicated that their facility had a formal policy on how to administer and use POLST. There was a non-statistically significant trend toward nursing homes in coalition areas having a POLST champion and formal policy compared to facilities in non-coalition areas.

Eighty-two percent of nursing homes reported that their staff had received education about POLST, and facilities reporting such education estimated that 43 % of staff had been educated. When education about POLST occurred, it nearly always included general orientation to the paradigm and form, and about three-fourths of the time included teaching the POLST conversation. However, case discussions and role play occurred in less than half of POLST educated nursing homes, and was more common in coalition areas than in non-coalition areas (53 % v 36 %, *p* = 0.01) (Table 2).

**Table 2:** Structural Factors and Education About POLST in Nursing Homes in California

	Overall	Coalition areas	Non-coalition areas	P- value^*^
**Structural**				
NH has a formal policy on POLST	59.2 %	65.1 %	53.7 %	0.06
NH has a POLST champion	70.4 %	75.6 %	65.0 %	0.07
NH has a place to put the POLST	98.5 %	100 %	96.9 %	0.07
**Staff education**				
SNF staff received POLST education	82.3 %	85.4 %	79.3 %	0.18
% NH staff received POLST education^†^	42.7 %	43.4 %	42.1 %	0.78
Types of education about POLST^†^				
General orientation	93.6 %	93.4 %	93.8 %	0.90
Teaching POLST conversation	75.5 %	76.3 %	74.6 %	0.76
Role play/case discussion	45.1 %	53.2 %	36.5 %	0.01
Written material	79.7 %	84.2 %	75.0 %	0.09

*POLST* Physicians Orders for Life Sustaining Treatment, *NH* nursing home

*P-value comparing nursing homes in coalition areas and non-coalition areas

^†^Among nursing homes reporting any staff receiving education

### POLST Use

**Figure 1.**
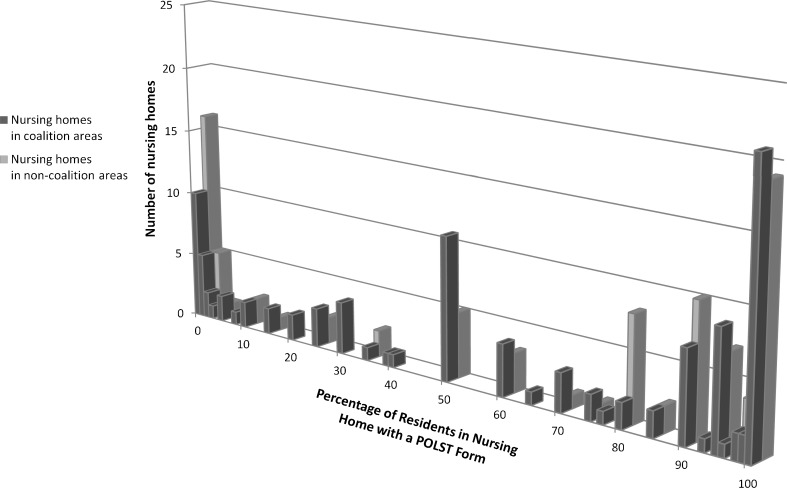
Histogram of nursing home residents that have a POLST form, comparing nursing homes in coalition and non-coalition areas. This Figure shows the percentage of residents in each nursing home that have a completed POLST form, and compares nursing homes in coalition and non-coalition areas, 2010.

Nearly 69 % of nursing homes reported that they had admitted a resident who had a POLST form completed, but there was not a statistically significant difference between nursing homes in coalition areas and non-coalition areas. Overall, nursing homes reported that 14.9 % of the residents admitted over the past 30 days had arrived with a completed POLST form, with no difference between coalition and non-coalition areas (18.4 % vs. 11.9 %, respectfully, *p* = 0.09). Eighty-one percent of nursing homes had administered a POLST with a patient. Overall, nearly 54 % of nursing home residents had a POLST. In coalition areas, more nursing home residents had a POLST than in non-coalition areas (59.8 % v 48.0 %, *p* = 0.02) (Table 3). The distribution of POLST use among nursing home residents is demonstrated in Fig. 1. There was a bimodal distribution of the proportion of nursing home residents who had completed a POLST, with 18 % in which no resident had done so and 27 % reporting that all residents had completed a POLST.

**Table 3:** Use of POLST in Nursing Homes in California

	Overall	Coalition areas	Non-coalition areas	P- value^*^
NH has admitted a resident with a POLST	68.6 %	72.3 %	65.0 %	0.19
% of residents admitted to the NH with a POLST	14.9 %	18.4 %	11.9 %	0.087
NH has completed a POLST with a resident after admission	80.9 %	84.0 %	77.9 %	0.21
% of residents in the NH who have a POLST	53.7 %	59.8 %	48.0 %	0.018

*POLST* Physicians Orders for Life Sustaining Treatment, *NH* nursing home

^*^ P-value comparing nursing homes in coalition areas and non-coalition areas

### Difficulties in Using POLST

Fewer than 10 % of nursing homes indicated that they experienced difficulties in following orders in a POLST, translating a POLST into nursing home orders, or having Emergency Medical Services follow POLST orders. However, 21 % of nursing homes had difficulty in interpreting a POLST to make treatment decisions, and more than one quarter reported that a family had disagreed with POLST content. More than one third of nursing homes reported difficulty in obtaining physician participation in POLST completion and having physicians sign the POLST. More than half of nursing homes reported difficulties in retrieving original POLST forms from other facilities. There were no differences in the frequency of reported problems between facilities in coalition areas and non-coalition areas (Table 4).

**Table 4:** Percentage of Nursing Homes Reporting Difficulties in Using the POLST

	Overall	Coalition areas	Non-coalition areas	P-value^*^
Translating POLST into NH orders	8.8 %	8.2 %	9.3 %	0.75
Interpreting POLST to make treatment decisions	20.8 %	22.4 %	19.1 %	0.51
Physician participation in POLST completion	37.7 %	38.5 %	36.9 %	0.79
Getting physician to sign POLST	34.2 %	36.1	32.3	0.52
Following orders in a POLST	6.5 %	7.7 %	5.4 %	0.45
Getting Emergency Medical Services to follow POLST orders	5.9 %	4.6 %	7.3 %	0.36
Retrieving original POLST from other facilities	62.1 %	65.9 %	58.1 %	0.20
Family disagreement with POLST content	28.1 %	26.9 %	29.4 %	0.66

*POLST* Physicians Orders for Life Sustaining Treatment, *NH* nursing home

^*^ P-value comparing nursing homes in coalition areas and non-coalition areas

### Level of Interaction Between Coalitions and Nursing Homes, and Relationship to Nursing Home POLST Structure and Use

Of the 143 nursing homes in coalition areas that responded to the survey, 75 were nursing homes with which the coalition reported working. Coalitions reported a level of interaction for 69 of these nursing homes: 12 were low, 32 moderate, and with 25 of the nursing homes, the level of interaction was high. Comparing survey responses of nursing homes that had any interaction with a coalition to nursing homes that had no interaction (Table 5, columns 2 and 3) showed that, in general, nursing homes with coalition interaction reported higher levels of POLST structure and use. This reached statistical significance for the percentage of nursing homes in which a POLST was completed with a resident (92.1 % in nursing homes with coalition interaction vs. 76.5 % in nursing homes without coalition interaction, *p* = 0.015). In addition, education of staff was more likely to employ case discussion and role play in nursing homes that had coalition interaction compared to those without (66.1 % v 38.5 %, respectively, *p* = 0.004). Statistically significant differences between nursing homes with low, moderate and high coalition interaction were seen for aspects of POLST education, but not POLST use (Table 5).

**Table 5:** Relationship Between Level of Interaction and Nursing Home POLST Structure and Use Among Nursing Homes in Coalition Areas

	Level of interaction between coalition and NH				
	None (*N* = 68)	Any (*N* = 69)	Low (*N* = 12)	Moderate (*N* = 32)	High (*N* = 25)
**Structural**					
NH has a formal policy on POLST	57.8 %	72.6 %	75.0 %	75.9 %	68.0 %
NH has a POLST champion	70.5 %	80.7 %	80.0 %	73.3 %	90.9 %
NH has a place to put the POLST	100 %	100 %	100 %	100 %	100 %
**Staff education**					
SNF staff received POLST education	80.6 %	90.0 %	83.3 %	90.6 %	92.3 %
% NH staff received POLST education	40.1	46.5	14.9	48.2	61.0†
Types of education about POLST					
General orientation	96.5 %	90.6 %	81.8 %	93.1 %	91.7 %
Teaching POLST conversation	71.2 %	80.7 %	63.6 %	81.5 %	87.5 %
Role play/case discussion	38.5 %	66.1 %*	45.5 %	60.0 %	82.6 %†
Written material	78.9 %	88.7 %	63.6 %	92.6 %	95.8 %†
**POLST use**					
NH has admitted a resident with a POLST	69.1 %	75.4 %	100 %	71.9 %	68.0 %
% of residents admitted to the NH with a POLST	14.0	22.7	16.0	20.0	28.4
NH completed a POLST with a resident after admission	76.5 %	92.1 %*	88.9 %	96.6 %	88.0 %
% of residents in the NH have a POLST	53.7	65.7	61.6	66.7	66.4

*POLST* Physicians Orders for Life Sustaining Treatment, *NH* nursing home

*P-value ≤ 0.05 for comparison between no interaction and any interaction groups

^†^ P-value < 0.05 for comparison across low, moderate and high interaction groups

## DISCUSSION

POLST is increasingly recognized as an important tool for involving patients in determining preferred level of aggressiveness of medical care, and an essential element in ensuring that appropriate care is provided to patients as they transition among community medical venues. This report of the early dissemination of POLST in California demonstrates widespread use of this public health intervention: after only 18 months, POLST was used in eight of ten nursing homes in this statewide sample, and two-thirds of nursing homes reported receiving a patient with a completed POLST from another care venue, suggesting use of POLST throughout the medical community. The higher rates of POLST use in coalition areas compared to facilities in non-coalition regions and the “dose response” seen in aspects of nursing home structure, education, and use with increasing coalition-nursing home interaction, suggests that the novel community-based dissemination mechanism employed in California is responsible for the rapid uptake of POLST within the state.

The community coalition model focused on creating materials for education about POLST, and implementation of policies and procedures to facilitate use of the documents. Uptake of these efforts was broad, with 82 % of nursing facilities reporting that their staff had received education about POLST, and most nursing homes having a POLST champion and policy. While the majority of nursing homes had used the POLST and many had a completed document for most residents, the heterogeneity of POLST use across nursing homes—as seen in Figure 1—was large, with 13 % of nursing homes having fewer than 10 % of residents with a completed POLST. Thus, although uptake was broad and rapid, there remains considerable room for improvement, particularly in non-coalition area nursing facilities, early in the course of implementation of this health intervention.

Community-based interventions to improve end-of-life care are not new, although none has used the model studied here. A statewide campaign in Hawaii to improve end-of-life care, “Kokua Mau,” aimed to bring together health care provider organizations, insurance companies, faith communities, policy makers and the public to increase advance directive use. The effort reached many people, but had only a modest effect on increasing advance directive completion.^15^ Efforts aimed at changing an individual community, such as the Respecting Choices program in La Crosse, Wisconsin, have been successful at changing practices within a relatively circumscribed, homogeneous community.^16^ The statewide community coalition effort undertaken in California across a large, heterogeneous population appears to be a novel effort that demonstrates the ability to disseminate a health intervention by facilitating local education and advocacy efforts. This model may have implications for states that are initiating POLST efforts.

This study demonstrated broad uptake of POLST after only a brief time, but also pointed out areas in which nursing homes identified improvement needs. While there was little difficulty in translating POLST information into care, more than one third of facilities noted difficulty in engaging physicians, which should be a focus of intervention. Furthermore, nursing homes noted that they had difficulty retrieving POLST documents that were transmitted elsewhere, suggesting that early in dissemination hospitals and other healthcare facilities may be less engaged in the use of POLST; study of POLST dissemination in other areas is needed.

This study has several limitations. The design aimed to obtain a statewide view of POLST use in California, while at the same time evaluating the effect of the community coalition intervention model. This meant that rural counties with few nursing homes were not included in the sampling frame, and that the findings cannot be generalized to such facilities. In addition, the response rate was low; the uptake of POLST reported by respondents may overstate actual penetration. During the study period, other influences, such as interventions from payers, may have affected POLST dissemination and we are unable to account for these. Furthermore, the findings reflect nursing home reported structural changes, education and POLST use; social desirability bias may compromise these data. The study design aimed to minimize these biases by involving a statewide trade organization and an independent evaluation team in order to enhance response rate and survey veracity.

This statewide evaluation of the early dissemination of POLST in California nursing homes shows broad use, suggesting promise for the novel community-based dissemination model. The survey also shows considerable heterogeneity in preparation for and use of POLST across nursing facilities, as might be expected for a new health intervention. Evaluation of POLST uptake in other aspects of healthcare, such as hospitals, is needed as is serial evaluation to study the pattern of further dissemination in nursing facilities. Most importantly, evaluation of the implications of POLST use on the medical care of Californians is needed.

## Electronic supplementary material

Below is the link to the electronic supplementary material.

ESM 1 (DOC 84 kb)
