# Supplementary material for: Implementation of Physician Orders for Life Sustaining Treatment in Nursing Homes in California: Evaluation of a Novel Statewide Dissemination Mechanism
Source: J Gen Intern Med. 2012 Aug 10;28(1):51–7. doi: 10.1007/s11606-012-2178-2 (PMC3539039; doi:10.1007/s11606-012-2178-2)
Supplement: Supplementary file 1 — (DOC 84 kb) [file 11606_2012_2178_MOESM1_ESM.doc]

**Survey about POLST**

1. Has your SNF ever admitted a resident who already had a completed POLST form?

Yes

No → ***Skip to question 4***

Don’t know → ***Skip to question 4***

2. Does your SNF have a specific place in the medical record to put the POLST?

Yes

No

Don’t know

3. Approximately what percentage of the residents admitted to your SNF in the past 30 days had a completed POLST form upon admission?

% Don’t know

4. Has a POLST form ever been completed for a resident after admission to your SNF?

Yes

No→ ***Skip to question 7***

Don’t know → ***Skip to question 7***

5. Who usually initiates discussion about POLST with the resident or resident’s family?

***Choose one answer*** Nurse Physician Social services

A dedicated team → Who? Other staff:

All different people initiate POLST documents

6. Is there a “champion” for POLST within the SNF, for example someone for whom advocating for POLST is part of their job responsibilities?

Yes→ What is this person’s job title? No

7. Approximately what percentage of the residents in your SNF currently have a POLST form?

% Don’t know

8. Does your SNF have a formal policy on how to complete and adhere to POLST forms?

Yes

No

Don’t know

9. Have SNF staff ever received education about POLST?

Yes

No → ***Skip to question 12***

10. What aspects of POLST education have been completed at your SNF?

***Respond Yes or No for each item***

a. General orientation about POLST

**Yes No**

b. Teaching about having the POLST conversation

c. Role play or case discussion about POLST

d. Distribution of written material about using POLST

Other:

11. Approximately what percentage of the SNF staff have received education about POLST?

% Don’t know

12. Has your SNF encountered any of the following problems with POLST?

***Respond Yes or No for each item***

**Yes No**

a. Translating the POLST into SNF medical record orders

b. Interpreting the POLST to make treatment decisions

c. Getting physicians to participate in completion of POLST

d. Getting physicians to sign a POLST form

e. Following the orders contained in the POLST

f. Getting EMS to follow the orders contained in the POLST

g. Receiving the original POLST back from other facilities

h. Family disagreement with POLST content

Other:

If “Yes” to any of the above, please describe:

a. Educational program for staff

Yes No

b. Example policy for handling POLST

Other:

Now a few questions about your views about POLST. For each of the following items please indicate if you Strongly agree, Agree, are Neutral, Disagree or Strongly disagree:

14. The value of POLST makes it worth the extra effort.

15. I feel confident that this SNF can honor a POLST ordering comfort care.

16. In this SNF, a POLST would be useful for many patients who do not have one.

Strongly

Agree Agree Neutral Disagree

Strongly

Disagree

Now some questions about your SNF

17. Approximately what proportion of total days are covered by the following insurance?

| Post acute Medicare |  | % |
| --- | --- | --- |
| Medicaid |  | % |
| Private pay |  | % |
| Other: |  | % |
|  |  | 1 0 0 % |

18. Over the past month, on average, what percent of your SNF beds were filled?

%

19. What is the primary language of the residents in your SNF?

| English |  | % |
| --- | --- | --- |
| Spanish |  | % |
| Other: |  | % |
| Other: |  | % |
|  |  | 1 0 0 % |

20. Does your SNF accept residents who are receiving care under the Medicare hospice benefit?

Yes

No

Don’t know

Who completed this survey? Name:

Title:

Telephone #: - -
